# Association between serum klotho levels and cardiovascular disease risk factors in older adults

**DOI:** 10.1186/s12872-022-02885-2

**Published:** 2022-10-11

**Authors:** Jaeho Lee, Donghoon Kim, Hyo-jung Lee, Ju-Young Choi, Jin-Young Min, Kyoung-Bok Min

**Affiliations:** 1 Department of Preventive Medicine, College of Medicine, Seoul National University, Seoul, 03080 Republic of Korea; 2 Integrated Major in Innovative Medical Science, Seoul National University Graduate School, Seoul, 03080 Republic of Korea; 3 Veterans Medical Research Institute, Veterans Health Service Medical Center, Jinhwangdo-ro 61-gil 53, Gangdong-gu, Seoul, 05368 Republic of Korea; 4 Institute of Health Policy and Management, Seoul National University Medical Research Center, Seoul, 03080 Republic of Korea; 5 Department of Preventive Medicine, College of Medicine, Seoul National University, 103 Daehak-ro, Jongno-gu, Seoul, 110799 Republic of Korea

**Keywords:** Dyslipidemia, Triglycerides, Klotho, Aging

## Abstract

**Background:**

Klotho deficiency is a significant predictor of cardiovascular disease (CVD)-related mortality and morbidity. However, research assessing the association between klotho and individual risk factors of CVD is limited. This study aimed to explore the association between circulating serum klotho levels and risk factors for CVD in adults.

**Methods:**

We used the 2007–2016 National Health and Nutrition Examination Survey and included 13,154 participants for whom serum klotho levels were available. Body mass index (BMI), exercise, smoking status, alcohol consumption, hypertension, dyslipidemia, serum lipid parameters, and blood pressure were considered as CVD risk factors.

**Results:**

Circulating klotho levels were negatively associated with being overweight (beta coefficient: − 22.609, *p* = 0.0025), obesity (beta coefficient: − 23.716, *p* = 0.0011), current smoking (beta coefficient: − 46.412, *p* < 0.0001), and alcohol consumption (beta coefficient: − 51.194, *p* < 0.0001). There was a positive association between serum klotho levels and no history of dyslipidemia (beta coefficient: 15.474, *p* = 0.0053). Serum klotho levels were significantly decreased by a unit increase in triglycerides (beta coefficient: − 0.117, *p* = 0.0006) and total cholesterol (beta coefficient: − 0.249, *p* = 0.0002). There was a significant non-linear relationship between serum klotho levels, triglycerides, and total cholesterol.

**Conclusions:**

Lower serum klotho levels are associated with certain CVD risk factors, including high BMI, smoking, alcohol consumption, and lipid parameters (triglycerides and total cholesterol). This study suggests that the soluble klotho level may be a potential marker for CVD risk.

## Background

Klotho encodes a transmembrane protein expressed in the kidney, parathyroid glands, and brain. It has also been observed in several other areas, such as reproductive organs, skeletal muscle, urinary bladder, reproductive glands, and vasculature [1–3]. There are two forms of klotho: membrane and soluble. Membrane klotho acts on the phosphatonin fibroblast growth factor (FGF)-23 [4], and a secreted klotho form of 70 kDa is generated by alternative RNA splicing, releasing the entire cleaved extracellular domain [5]. The soluble form has been detected in urine, blood, and cerebrospinal fluid due to the shedding of the transmembrane form by different cellular surface metalloproteases [5, 6].

Recent studies have focused on klotho, initially identified as an anti-aging suppressor gene in mice [1]. Klotho encodes a single-pass transmembrane protein expressed in the kidneys, parathyroid glands, and brain [1]. Although the biological mechanism of the klotho protein is not well understood, its pleiotropic functions include inhibition of insulin/insulin-like growth factor 1 (IGF-1), regulation of energy and mineral metabolism, and suppression of oxidative stress and inflammatory responses [1, 7]. Klotho deficiency or aberrant klotho expression has been observed in several age-related disorders, including chronic kidney disease, cancer, and diabetes. Accumulating evidence suggests that klotho plays a protective role in the vascular system by maintaining endothelial homeostasis and vascular functionality and preventing vascular calcification [4, 5, 8].

Klotho appears to exert a protective effect on the vessel wall [9–13]. A functional variant of klotho is an independent risk factor for early onset coronary artery disease (CAD), ischemic stroke, and atherosclerotic CAD [9–13]. Reduced soluble klotho levels are associated with an increased CVD mortality rate in hemodialysis patients and vascular dysfunction in patients with kidney disease patients [10, 11]. In a population-based study, serum klotho levels were inversely associated with the probability of having CVD [12] or increased brachial artery and epicardial fat thickness [13].

Although the studies mentioned above determined klotho deficiency as an important factor for predicting CVD and mortality, the current understanding of CVD risk factors associated with circulating serum klotho levels, such as smoking and obesity, is limited. Given that a low klotho level is associated with a high risk of CVD, we hypothesized that the significant risk factors for CVD are associated with low serum klotho levels. Therefore, this study aimed to examine the association between serum klotho levels and well-established risk factors for CVD, including smoking, alcohol consumption, obesity, cholesterol levels, physical inactivity, and chronic diseases.

## Methods

### Study population

We extracted patient data from the NHANES for the years 2007–2016. The NHANES, conducted by the Centers for Disease Control and Prevention, is a nationally representative survey of the noninstitutionalized civilian population in the United States. From the 2007–2016 NHANES data, 17,385 participants aged 40–79 years were initially included. We then selected 13,763 respondents who agreed for their klotho samples to be used. Patients with missing data were excluded from the study. Finally, 13,154 individuals were selected as study participants. The participants provided oral and written informed consent before the survey. The National Center approved the study protocol for the Health Statistics Institutional Review Board.

### Measurement of serum klotho

Serum samples were collected from the 2007–2016 NHANES laboratory data. An ELISA kit (IBL International, Japan) was used to validate serum concentrations in human samples. The validation results informed research inspectors. The Northwest Lipid Metabolism and Diabetes Research Laboratories, Division of Metabolism, Endocrinology, and Nutrition at the University of Washington, conducted analyses of serum specimens, except for four fresh frozen samples. The samples were secured on a dry ice package and monitored by laboratory personnel. Batches of samples were stored at − 80 °C and analyzed daily by the technicians in duplicate. The average of the two concentrations was used to determine the final values. Two quality control samples containing low and high concentrations of klotho were analyzed in duplicate on each plate. The results of the analyses were transported from the instrument to the laboratory Oracle Management System for evaluation. Duplicate values of samples greater than 10% were designated as repeated analyses. The entire plate was reproduced when the value of a quality control sample deviated from the range of two standard deviations of the assigned value [14].

### Variables of interest

The demographic variables of interest included age, sex (male or female), race/ethnicity (Mexican American, other Hispanic, non-Hispanic white, non-Hispanic black, Asian), income (< $45,000 or ≥ $45,000), and education (non-high school graduate, high school graduate, or college graduate or over).

Body mass index (BMI), calculated by dividing an individual’s weight by his or her height squared, was treated as a continuous variable. Then, BMI was categorized into the following four groups: underweight (< 18.5 kg/m^2^), normal weight (18.5–22.9 kg/m^2^), overweight (23–24.9 kg/m^2^), and obese (≥ 25 kg/m^2^). The exercise was divided into regular and non-exercise-based groups. Smoking status was classified as current, former, or never smoker. Participants who had at least 12 drinks of any type of alcoholic beverage per year were considered as drinkers. Hypertension was defined as high diastolic blood pressure (≥ 90 mmHg) or systolic blood pressure (≥ 140 mmHg). Dyslipidemia was defined as any one of the following conditions: total cholesterol ≥ 240 mg/dL, triglycerides > 200 mg/dL, low-density lipoprotein (LDL) cholesterol ≥ 160 mg/dL, and high-density lipoprotein (HDL) cholesterol < 40 mg/dL.

### Statistical methods

We used one-way ANOVA for ordinarily continuous variables and chi-square tests for categorical variables to compare participants’ characteristics according to the quartile of serum klotho. Continuous variables are presented as means and standard deviations, and categorical variables are presented as numbers and percentages. Multivariate regression models were used to determine the relationship between serum klotho levels and CVD risk factors with the estimated beta values and standard error (SE). The regression model was adjusted for age, sex, ethnicity, education, and income as covariates. The models were further stratified according to sex. Additionally, to explain the nonlinear relationship between serum klotho and CVD risk factors, a generalized additive model (GAM) was used. The GAM provided a serum klotho’ estimated smoothing spline function of serum klotho with a 95% confidence interval (95% CI) when CVD risk factors were considered as independent variables.

We used weighted estimates of population parameters based on the National Health and NHANES analytic and reporting guidelines. All analyses were performed using PROC SURVEY and PROC GAM procedures in SAS 9.2 (SAS Institute, Cary, NC, USA) to explain the complex sampling scheme. All tests were two-sided, and the level of statistical significance was set at α = 0.05.

## Results

Table 1 summarizes the patient demographic characteristics. A total of 13,154 individuals were included in the analysis and divided into four groups based on serum klotho quartiles. The mean age of the participants was approximately 58 years, and 46.06% of the participants were male. Of the participants, 48.81% were of other races, and there were relatively few Asians (6.24%). The proportion of age and level of education in high school decreased across the serum klotho quartiles.

**Table 1:** Demographics of Participants characteristics according to quartile of plasma klotho. Values are mean ± SD or n (%)

Plasma klotho (pg/ml)	< 654.6	654.6 to 802.4	802.4 to 993.3	$\ge$ 992.4	*p* value^a^
N	3288	3286	3291	3289	
Age (years)	59.10 ± 11.11	57.94 ± 10.84	57.35 ± 10.73	56.35 ± 10.62	< .0001
Sex					< .0001
Male	1669 (26.32%)	1704 (26.87%)	1554 (24.50%)	1414 (22.29%)	
Female	1589 (23.42%)	1582 (23.32%)	1737 (25.60%)	1875 (27.64%)	
Race/ethnicity					< .0001
Mexican American	250 (23.94%)	262 (25.09%)	287 (27.49%)	245 (23.46%)	
White	778 (26.35%)	793 (26.86%)	755 (25.57%)	626 (21.20%)	
Black	393 (24.88%)	329 (20.83%)	347 (21.97%)	510 (32.29%)	
Asian	177 (20.60%)	228 (26.54%)	234 (27.24%)	220 (25.61%)	
Other race	1690 (25.14%)	1674 (24.91%)	1668 (24.82%)	1688 (25.11%)	
Income					0.9008
Low	1776(25.84%)	1689(24.57%)	1710(24.87%)	1698(24.70%)	
High	1512(24.07%)	1597(25.42%)	1581(25.17%)	1591(25.33%)	
Education					0.3950
Less than high school	949 (26.08%)	906 (24.90%)	888 (24.40%)	895 (24.60%)	
High school	792 (27.23%)	700 (24.07%)	740 (25.44%)	676 (23.24%)	
College or more	1547 (23.41%)	1680 (25.42%)	1663 (25.16%)	1718 (25.99%)	

Income: low (Total annual family income < $45,000) and high (Total annual family income ≥ $45,000)

^a^The *p* value is based on the T-test for binominal groups and Wald F-test for categorical groups

**Fig. 1:**
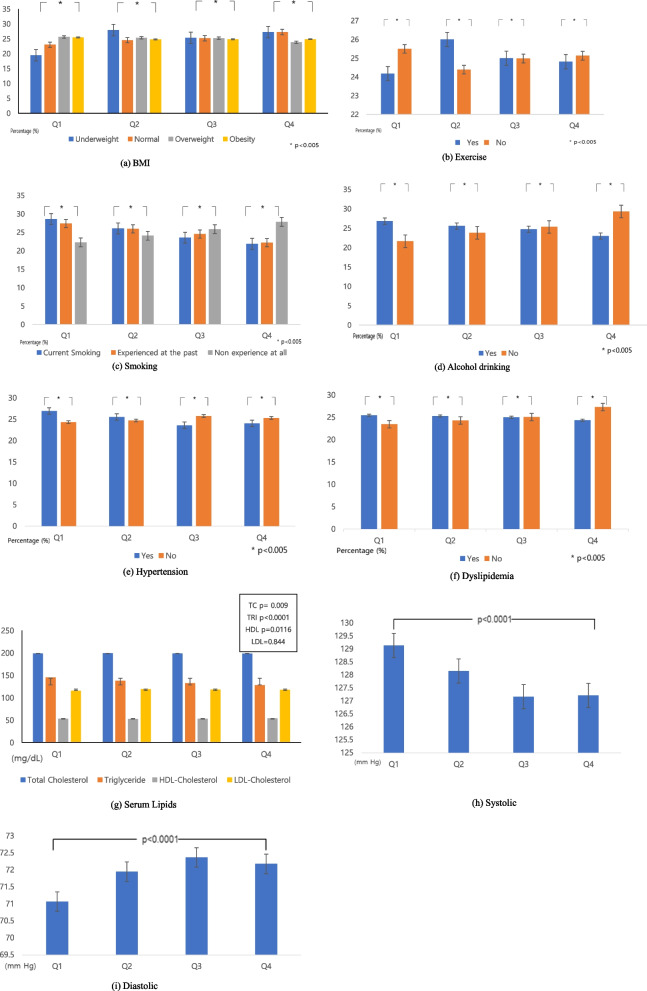
The Distribution of CVD risk factors by the plasma klotho (pg/ml) quartile using a bar graph: **a** BMI, **b** Exercise, **c** Smoking, **d** Alcohol drinking, **e** Hypertension, **f** Dyslipidemia, **g** Serum Lipids, **h** Systolic Blood Pressure, and **i** Diastolic Blood Pressure

Figure 1 shows the distribution of CVD risk factors according to the klotho quartiles. There were significant associations between CVD risk factors and serum klotho quartiles. Participants in the lowest quartile (Q1) had lower normal BMI, exercise, HDL, and diastolic blood pressure than those in the highest quartile (Q4). Specifically, the proportion of participants with normal BMI and HDL increased gradually as the quantile reached the highest quartile (Q4). There was no significant association with LDL levels (*p* = 0.844) among the four quartiles. In contrast, participants in the lowest quartile (Q1) were more likely to be overweight/obese, smokers, and alcohol drinkers. They were also more likely to have hypertension, dyslipidemia, elevated triglyceride levels, and elevated systolic blood pressure. The proportion of participants with obesity, smoking, alcohol drinking, dyslipidemia, and triglycerides gradually decreased as the quantile increased (Q4).

Table 2 presents the association between CVD risk factors and klotho in the total study population, stratified by sex using multivariate regression analysis. The analysis of overweight (beta coefficient: − 29.258) and obesity (beta coefficient: − 22.941) with serum klotho showed a negative association. After adjusting for age, sex, ethnicity, education, and income, the association maintained a significant negative relationship (beta coefficient: − 22.607, − 23.716). There were no significant associations between serum klotho levels and exercise, LDL, or diastolic blood pressure. Analysis of smoking (beta coefficient: − 50.666) and alcohol consumption (beta coefficient: − 56.952) showed a negative relationship between serum klotho levels. The reference group was designated as non-smokers and non-alcohol drinkers. After adjustment, the association maintained a strong relationship between smoking (beta coefficient: − 46.412) and alcohol consumption (beta coefficient: − 51.194). Serum klotho levels showed a positive relationship with hypertension (beta coefficient: 17.371) and dyslipidemia (beta coefficient: 22.075) in individuals with hypertension or dyslipidemia. After adjustment, the association of dyslipidemia (beta coefficient: 15.474) with serum klotho was still significant; however, hypertension showed no significant relationship. Triglycerides (beta coefficient: − 0.146) and total cholesterol (beta coefficient: − 0.161) levels were negatively correlated with serum klotho levels. HDL showed a positive relationship (beta coefficient: 0.397) with serum klotho levels; however, the results from the adjusted model were insignificant. Systolic blood pressure was negatively associated with serum klotho levels (beta coefficient: − 0.624); in contrast, the association was insignificant after adjusting the model. Stratification of serum klotho by sex showed a positive relationship with hypertension (beta coefficient: 17.588) in males and dyslipidemia (beta coefficient:37.511) in females. Serum klotho levels showed a negative relationship with smoking, alcohol consumption, and total cholesterol levels in both sexes. The analyses of overweight (beta coefficient: − 26.068), obesity (beta coefficient: − 38.948), and triglycerides (beta coefficient: − 0.285) showed a negative relationship only in females. HDL showed a negative relationship with serum klotho (beta coefficient: − 0.654) in males but showed a positive relationship (beta coefficient: 0.499) in females.

**Table 2:** Multivariate regression analysis of the association between CVD risk factors and Plasma Klotho

Plasma klotho (pg/ml)	Total population		Stratification by gender			
			Male		Female	
	Beta coefficient^a^ (SE)	*p* value	Beta coefficient^a^ (SE)	*p* value	Beta coefficient^a^ (SE)	*p* value
BMI						
Underweight	12.798 (26.550)	0.6298	− 4.644 (38.501)	0.9040	18.798 (36.491)	0.6065
Normal weight	Reference				Reference	
Overweight	− 22.607 (7.483)	0.0025	− 19.077 (10.237)	0.0624	− 26.068 (11.322)	0.0213
Obesity	− 23.716 (7.264)	0.0011	− 12.607 (10.779)	0.2422	− 38.948 (10.506)	0.0002
Exercise						
Yes	Reference				Reference	
No	− 3.691 (5.549)	0.5059	9.663 (7.370)	0.1898	− 16.763 (8.194)	0.0408
Smoking						
Current smoking	− 46.412 (7.512)	< .0001	− 34.831 (9.830)	0.0004	− 60.661 (11.426)	< .0001
Experienced at the past	− 24.207 (6.444)	0.0002	− 24.461 (8.338)	0.0034	− 26.961 (9.883)	0.0064
None	Reference				Reference	
Alcohol drinking						
Yes	− 51.194 (6.768)	< .0001	− 49.196 (10.666)	< .0001	− 55.834 (8.746)	< .0001
No	Reference				Reference	
Hypertension						
Yes	Reference				Reference	
No	4.767 (6.550)	0.4668	17.588 (8.430)	0.037	− 10.358 (10.095)	0.3049
Dyslipidemia						
Yes	Reference				Reference	
No	15.474 (5.546)	0.0053	− 4.570 (7.340)	0.5335	37.511 (8.361)	< .0001
1-unit increase in lipid profiles (mg/dL)						
HDL	0.010 (0.182)	0.9562	− 0.654 (0.272)	0.0162	0.499 (0.250)	0.0461
LDL	− 0.055 (0.117)	0.6409	0.069 (0.151)	0.6469	− 0.153 (0.176)	0.3845
TG	− 0.117 (0.033)	0.0006	0.052 (0.046)	0.2559	− 0.285 (0.077)	0.0002
Total Chol	− 0.249 (0.066)	0.0002	− 0.173 (0.087)	0.0479	− 0.315 (0.100)	0.0018
1-unit increase in blood Pressure (mm Hg)						
Systolic blood pressure	− 0.206 (0.158)	0.1934	− 0.267 (0.209)	0.2010	− 0.113 (0.234)	0.6276
Diastolic blood pressure	− 0.040 (0.287)	0.8891	− 0.089 (0.456)	0.8437	0.030 (0.351)	0.9308

^a^Adjusted for age, gender, ethnicity, education, and income

**Fig. 2:**
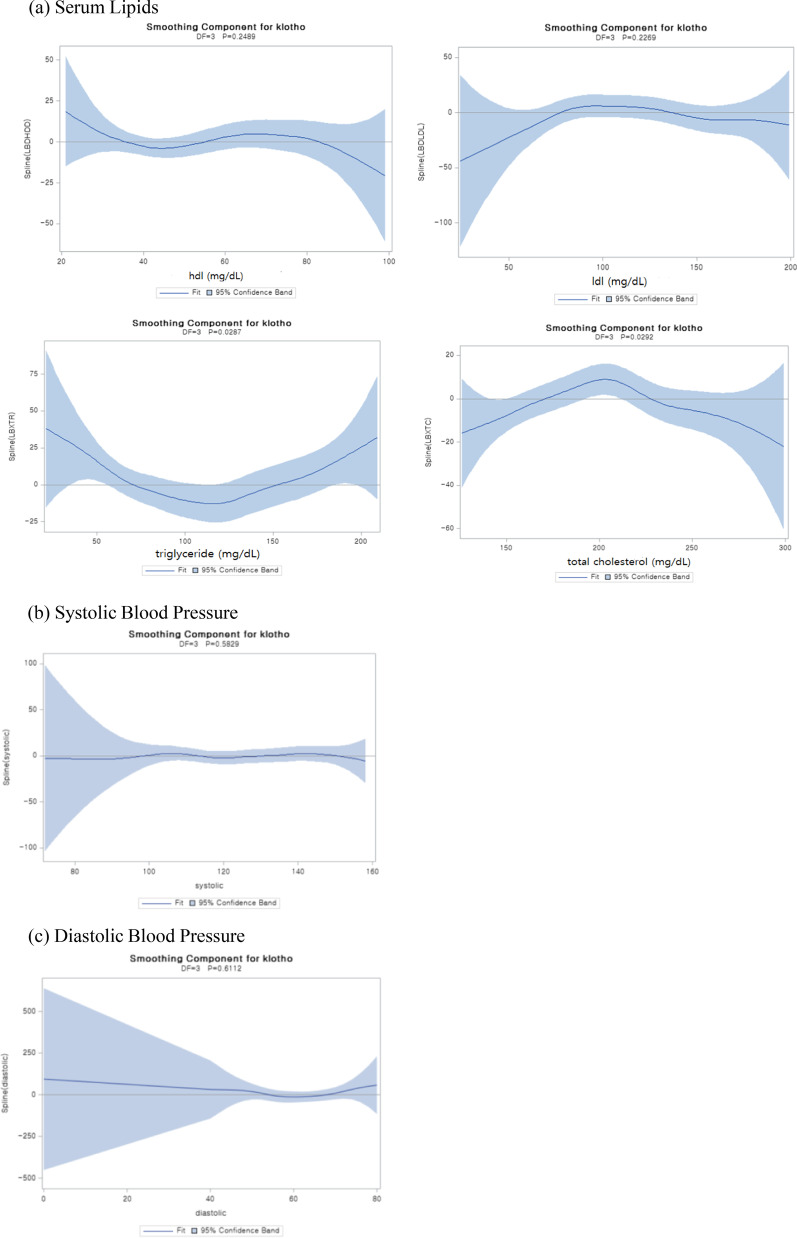
Plots of estimated smoothing spline function of serum klotho (pg/ml) and CVD risk factors for continuous variables. Shaded areas indicate a 95% CI for GAM. **a** Serum lipids, **b** systolic blood pressure, and **c** diastolic blood pressure

Figure 2 shows serum klotho’s estimated smoothing spline function with a 95% confidence band for the GAM when CVD risk factors were considered as independent variables. As expected from the results in Table 2, the estimated smoothing spline function of the GAM only showed a significant relationship with triglycerides (*p* = 0.0287) and total cholesterol (*p* = 0.0292). Our smoothing spline function suggests that individuals with lower triglyceride levels tend to have higher serum klotho levels than those with higher levels. Additionally, individuals with higher total cholesterol levels tended to have lower serum klotho levels.

## Discussion

Using nationally representative survey data from US adults, we investigated the association between serum klotho levels and certain cardiovascular risk factors such as smoking, alcohol consumption, obesity, cholesterol levels, physical inactivity, and chronic diseases. We found that adults with a higher risk of CVD had lower serum klotho concentrations; the lower the serum klotho level, the higher the rates of obesity, smoking, alcohol consumption, and higher levels of total cholesterol and TG. This association remained robust, despite adjusting for age, sex, ethnicity, education, and income. Our findings suggest that certain CVD risk factors may be associated with lower klotho levels, and controlling modifiable factors may help prevent or alleviate klotho decline.

The mechanism underlying this observation remains unclear, and understanding the biological role of klotho in the inflammatory response and oxidative stress may be key to elucidating this mechanism [15]. The Klotho protein promotes an anti-oxidative response via augmented production of superoxide dismutase or reduced generation of reactive oxygen species [15]. Klotho interacts with inflammatory mediators that modulate gene expression [16, 17] Thus, klotho deficiency may induce pro-oxidative and proinflammatory effects and show the association with CVD risk. Based on this reasoning, several CVD risk factors that we presented in this study (smoking, alcohol consumption, elevated lipid levels, and high BMI) may have a relevance with the modulation of inflammation and oxidative stress [18–21].

Smoking is a significant risk factor for CVD, activates leukocytes to release reactive oxygen and nitrogen species, and promotes the secretion of proinflammatory cytokines [22].Lam-Rachlin et al. (2013) reported a relationship between smoking and decreased serum klotho levels [23]. Among pregnant women with microbial invasion of the intra-amniotic cavity, smokers had lower serum klotho levels than non-smokers [23]. Studies examining alcohol consumption and klotho are scarce; however, a recent study reported that alcohol consumption negatively affects klotho levels [24]. Alcohol consumption is accompanied by increased inflammatory responses (IL-6, TNF-α, and C-reactive protein) and oxidative stress (malondialdehyde and 8-isoprostane) [18]. Thus, it is possible that behavioral CVD risk factors, such as smoking and alcohol consumption, decrease the expression levels of klotho protein via increased oxidative stress and inflammation.

Elevated BMI, both overweight and obese, is associated with dysregulated body weight homeostasis and is an established CVD risk factor [25]. High BMI/obesity is attributable to the secretion of inflammatory mediators, such as TNF-α and IL-6, and a reduction in adiponectin, predisposing the inflammatory response and oxidative stress [26, 27].The association between BMI and klotho in humans remains unclear; however, it was recently examined in a study by Landry et al. [28]. The authors found that overweight/obese subjects had significantly lower klotho levels in the cerebrospinal fluid than their lean counterparts [28]. In an experimental model, klotho-treated mice experienced reduced adiposity, increased lean mass, and high energy expenditure [29]. Considering that the role of klotho in regulating energy metabolism and angiogenesis is complex and uncertain, studies on whether klotho affects fat accumulation and whether BMI reduces klotho levels via increased inflammatory response or oxidative stress are needed.

Lipid parameters, including total cholesterol, HDL-cholesterol, LDL-cholesterol, and triglyceride levels, are strong and independent risk factors for CVD Studies assessing the direct association between serum lipid parameters and klotho levels in humans are rare. Kim et al. (2019) investigated the association between serum klotho levels and metabolic syndrome and found that hypertriglyceridemia was independently associated with reduced serum klotho levels [30]. Consistent with this, we found a significant association between lipid parameters and klotho levels, which was predominant in females. The mechanism underlying sex differences in the association between lipid profiles and klotho levels remains unclear. However, since sex hormones are important regulators of lipid kinetics and are responsible for sexual dimorphism in the lipid profile, the association between lipid and klotho levels may be affected differently according to sex. Further studies are needed to understand the potential link between lipid parameters and klotho levels and their sex differences.

The strengths of our study include the evaluation of multiple traditional CVD risk factors to verify their associations with serum klotho levels using public data from the NHANES. Moreover, we reported a population-based sample of adults with circulating klotho levels, in which most previous studies were conducted using genetic variants of klotho or animal studies. However, our study has some limitations. First, it only considered a cross-sectional design, which allowed us to demonstrate the association between soluble klotho levels and CVD risk factors. Therefore, it was difficult to explain the causality of these relationships. Future longitudinal studies considering time relationships should quickly examine the clinical importance of klotho for therapeutic interventions regarding CVD risk factors. Second, the characteristics of serum klotho are affected by circadian variations [16]. Circulating klotho levels at midnight decreased by approximately 40% from baseline and gradually returned to near-normal levels [16]. Third, although demographic variables were considered as confounding factors in our study, other potential confounding factors may still exist because of the inherent bias of the cross-sectional study design.

## Conclusions

We found that lower serum klotho levels are associated with certain CVD risk factors. Further studies are warranted to confirm the reliability of our findings, using a large number of datasets to validate our findings in an independent study population. Our study suggests that the soluble klotho level may be a potential therapeutic marker for CVD prevention.

## Data Availability

The NHANES data is publically available and can be downloaded from the following sites (https://wwwn.cdc.gov/nchs/nhanes/continuousnhanes/default.aspx?BeginYear=2007; https://wwwn.cdc.gov/nchs/nhanes/continuousnhanes/default.aspx?BeginYear=2009; https://wwwn.cdc.gov/nchs/nhanes/continuousnhanes/default.aspx?BeginYear=2011; https://wwwn.cdc.gov/nchs/nhanes/continuousnhanes/default.aspx?BeginYear=2013; https://wwwn.cdc.gov/nchs/nhanes/continuousnhanes/default.aspx?BeginYear=2015).

## Abbreviations

BMI: Body mass index
CAD: Coronary artery disease
CVD: Cardiovascular diseases
GAM: Generalized additive models
HDL: High density lipoprotein
LDL: Low density lipoprotein
TC: Total cholesterol
TG: Triglycerides
